# Evaluating the influence of common antibiotics on the efficacy of a recombinant immunotoxin in tissue culture

**DOI:** 10.1186/s13104-019-4337-6

**Published:** 2019-05-27

**Authors:** Yuyi Zhu, John E. Weldon

**Affiliations:** Department of Biological Sciences, The Jess and Mildred Fisher College of Science and Mathematics, Towson University, Towson, MD 21252 USA

**Keywords:** Recombinant immunotoxins, Combination therapy, Antibiotics, HB21-LR, *Pseudomonas* exotoxin A, Cytotoxicity, Mitochondrial translation, Translation inhibition

## Abstract

**Objective:**

Recombinant immunotoxins (RITs) are antibody-toxin fusion proteins that can selectively eliminate populations of cells expressing specific surface receptors. They are in evaluation as therapeutic agents for cancer. RITs based on *Pseudomonas* exotoxin A (PE) are in use clinically for the treatment of hairy cell leukemia, and under trial for the treatment of other cancers. In an effort to improve the efficacy of PE-based RITs, we evaluated the potential of combination therapy with several common antibiotics (tetracycline, chloramphenicol, streptomycin, linezolid, fusidic acid, and kanamycin) on human cell lines HEK293, OVCAR8, and CA46. Antibiotics were selected based on their potential to inhibit mitochondrial protein synthesis and disrupt energy metabolism in cancer cells.

**Results:**

Tetracycline, chloramphenicol, linezolid, and fusidic acid alone killed cultured human cells at high concentrations. At high but nontoxic concentrations of each antibiotic, only chloramphenicol treatment of the Burkitt’s lymphoma cell line CA46 showed enhanced cytotoxicity when paired with an anti-transferrin receptor/PE RIT. This result, however, could not be replicated in additional Burkitt’s lymphoma cell lines Ramos and Raji. Although the six antibiotics we tested are not promising candidates for RIT combination therapy, we suggest that fusidic acid could be considered independently as a potential cancer therapeutic.

**Electronic supplementary material:**

The online version of this article (10.1186/s13104-019-4337-6) contains supplementary material, which is available to authorized users.

## Introduction

Recombinant immunotoxins (RITs) are genetically engineered, chimeric proteins comprised of an antibody joined to a cytotoxic protein [1–3]. They are most commonly utilized as targeted therapeutics for the treatment of cancer, but are also in development as antiviral therapies [4]. The antibody binds to specific cell surface receptors and delivers its toxic payload in a targeted manner. Familiar toxins such as diphtheria toxin [5] and ricin [6] have been engineered for inclusion in RITs, but one of the most extensively utilized toxins is *Pseudomonas* exotoxin A (PE) [1]. Several PE-based RITs have been developed and brought to clinical trial [7], and one, moxetumomab pasudotox (Lumoxiti™, AstraZeneca), has been FDA-approved for the treatment of hairy cell leukemia [8]. Although PE RITs have great promise, most remain under development because of difficulties with nonspecific toxicity, immunogenicity, and poor activity against some cancers [1].

Recent improvements to the design and construction of PE RITs have reduced their immunogenicity and off-target effects while substantially retaining activity [9]. The cytotoxicity of RITs is now viewed as a key characteristic for improvement. Combination therapies of RITs with different chemical agents have been utilized to enhance their efficacy [10–12], but additional research into combination treatments is needed.

Dysregulated bioenergetics have been identified as a hallmark of cancer [13]. One potential avenue for RIT combination therapy is to disrupt the bioenergetic pathways of cancer cells by targeting mitochondria. Since mitochondria retain protein synthesis machinery similar to that of bacteria [14], antibiotics targeting bacterial translation can also inhibit mitochondrial translation [15, 16].

In this study, we attempted to determine if a combination therapy of PE-based RITs and antibiotics targeting bacterial protein synthesis might exhibit enhanced cytotoxicity. We tested FDA-approved antibiotics tetracycline, chloramphenicol, streptomycin, linezolid, fusidic acid, and kanamycin. Alone, tetracycline, chloramphenicol, linezolid, and fusidic acid killed cultured human cells at high concentrations. High, nontoxic concentrations of most antibiotics did not enhance the activity of the HB21-LR RIT, an anti-transferrin receptor scFv combined with PE24 [17, 18]. Only the combination of chloramphenicol and HB21-LR targeted against the CA46 Burkitt’s lymphoma cell line demonstrated enhanced cytotoxicity, but this effect could not be replicated on other Burkitt’s lymphoma cell lines. We conclude that these six antibiotics are not promising candidates for combination therapies with PE-based RITs, but fusidic acid could be considered individually for use as a cancer therapeutic.

## Main text

### Methods

#### Cell lines

Human-derived cell lines HEK293 (embryonic kidney), OVCAR8 (ovarian serous adenocarcinoma), and the Burkitt’s lymphoma cell lines CA46, Raji, and Ramos were grown in culture at 5% CO_2_ and 37 °C in DMEM with 4.5 g/l glucose, 1 mM sodium pyruvate, 10% FBS, and 2 mM l-glutamine. All cells were thawed and grown from liquid nitrogen stocks prepared from an early passage of the original cell lines obtained as a kind gift from the laboratory of Dr. Ira Pastan (NIH, Bethesda, MD). All cell lines evaluated are sensitive to HB21-LR.

#### Antibiotics

Six antibiotics were selected in this study: chloramphenicol, tetracycline, fusidic acid, kanamycin, linezolid, and streptomycin. Chloramphenicol (VWR Chemicals, Sanborn, NY) stocks were prepared in 200 proof ethanol to a concentration of 154.4 mM. Fusidic acid (Chem-Impex International, Wood Dale, IL) stocks were prepared in 200 proof ethanol to a concentration of 96.8 mM. Linezolid (Chem-Impex International, Wood Dale, IL) stocks were prepared in DMSO to a concentration of 59.3 mM. Kanamycin (VWR Chemicals, Sanborn, NY) stocks were prepared in ultrapure water to a concentration of 85.8 mM. Streptomycin (Thermo Fisher Scientific, Fair Lawn, NJ) stocks were prepared in ultrapure water to a concentration 34.3 mM. Tetracycline (Thermo Fisher Scientific, Fair Lawn, NJ) stocks were prepared in ultrapure water to a concentration of 104 mM. Prepared stocks were sterile filtered through a 0.22 µm syringe filter.

#### Recombinant immunotoxin

The anti-transferrin receptor/PE24 RIT HB21-LR was prepared as described [19], diluted in culture medium to a concentration of 10 μg/ml, aliquoted into single-use 50 μl aliquots, and stored at − 80 °C until used.

#### Cytotoxicity assays

Cell viability was evaluated using the WST-8 reagent (CCK-8, Dojindo Molecular Technologies, Inc., Gaithersburg, MD). Assays were performed essentially as described [20]. The 0% viability control was culture medium without cells and the 100% viability control was untreated cells in culture medium. Combination treatments were performed with a single nontoxic concentration of antibiotic (Table 1) and threefold serial dilutions of HB21-LR starting at a maximum concentration of 20 ng/ml. Data were analyzed using GraphPad Prism (GraphPad Software, Inc., La Jolla, CA) by fitting the results to a four-parameter sigmoid function and interpolating the concentration that resulted in 50% cell viability (EC_50_). A two-tailed paired T-test was utilized to evaluate significant differences in EC_50_ values by comparing experimental conditions to a control plate not treated with antibiotic. P values less than 0.01 were considered significant.

**Table 1:** Concentrations (µM) of antibiotics used in combination assays

Antibiotic	HEK293	OVCAR8	CA46	Ramos	Raji
Chloramphenicol	150	60	500	50	25
Tetracycline	50	100	100	–	–
Fusidic acid	50	50	10	–	–
Kanamycin	333	300	100	–	–
Linezolid	100	170	150	–	–
Streptomycin	100	100	100	–	–

### Results

#### Selection of antibiotics

Common antibiotics that inhibit bacterial translation are known to affect mitochondrial protein synthesis [15], and might even be repurposed to treat cancers [16]. We selected six antibiotics that have been tested in mammalian cells and are known to target components of mitochondrial protein synthesis. The chosen antibiotics were tetracycline [15, 16, 21, 22], chloramphenicol [23], streptomycin [21], linezolid [23], fusidic acid [15], and kanamycin [22].

#### Antibiotic cytotoxicity assays

Human cell lines HEK293, OVCAR8, and CA46 were evaluated for cytotoxic responses to the six antibiotics at least twice to determine a high but nontoxic dose of the antibiotic. Example viability responses are shown for HEK293 (Additional file 1), OVCAR8 (Additional file 2), and CA46 (Additional file 3). Neither streptomycin nor kanamycin showed a cytotoxic effect in any cell line. Both chloramphenicol and fusidic acid showed a measurable decrease in viability for all three cell lines. Additional Burkitt’s lymphoma lines Raji and Ramos, evaluated only for response to chloramphenicol, were also sensitive (Additional file 4). Tetracycline and linezolid showed variable effects. Tetracycline demonstrated toxicity at high concentrations in HEK293 and OVCAR8 cells, but was nontoxic in CA46. Linezolid was toxic at high concentrations in HEK293 cells, but was nontoxic in OVCAR8 and CA46. Estimated EC_50_ values for cell lines sensitive to antibiotics are shown in Additional file 5. The maximum concentration of each antibiotic evaluated is shown in Additional file 6. From these assays, high but nontoxic concentrations of antibiotics were selected for combination treatment (Table 1).

#### Combination cytotoxicity assays

**Fig. 1:**
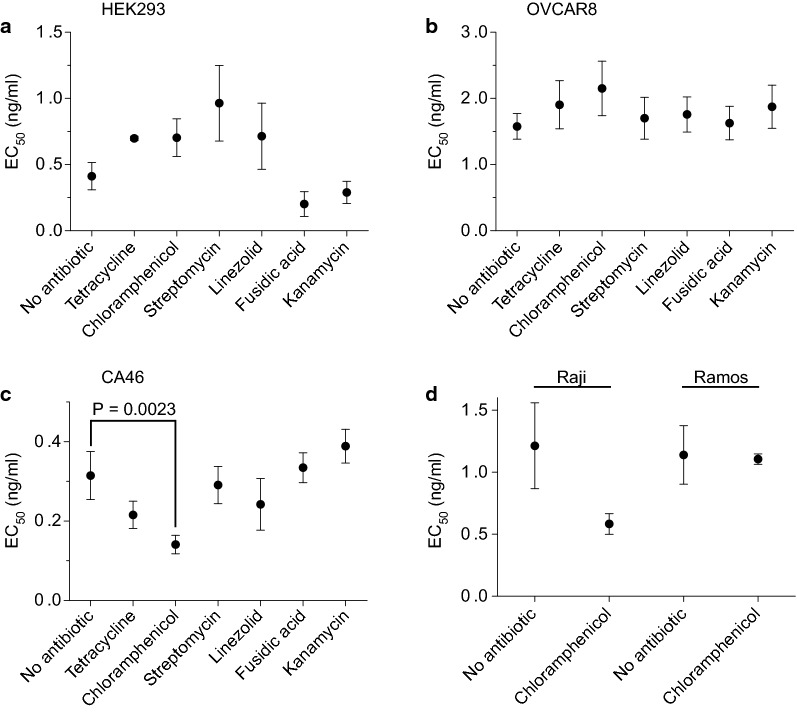
Responses of human cell lines to antibiotic combination treatment. Cytotoxicity assays were performed evaluating human cell lines HEK293, CA46, and OVCAR8 in response to the recombinant immunotoxin HB21-LR in the presence and absence of various antibiotics. EC_50_ values of each treatment condition are plotted. Error bars indicate standard error of the mean. All values are the average of at least three separate experiments. The P value shown is from a two-tailed, paired T test and reflects a significant difference between EC_50_ values of an antibiotic-treated and untreated control. Cell lines HEK293 (**a**), OVCAR8 (**b**), and CA46 (**c**) were evaluated in combination with antibiotics chloramphenicol, tetracycline, fusidic acid, kanamycin, linezolid, and streptomycin. Cell lines Ramos (**d**) and Raji (**d**) were evaluated in combination with chloramphenicol

Human cell lines were evaluated for cytotoxic response to HB21-LR in the presence of high but nontoxic doses of the six antibiotics. The results for HEK293, OVCAR8, and CA46 are shown in Fig. 1a–c. Representative cytotoxicity assays are shown in Additional files 7, 8, 9, 10. No significant difference was observed between antibiotic-treated cells and the untreated control in HEK293 and OVCAR8. Only the presence of chloramphenicol demonstrated an enhanced EC_50_ when compared to the untreated control in CA46 Burkitt’s lymphoma cells (P = 0.0023). Chloramphenicol was further evaluated on the Burkitt’s lymphoma cell lines Ramos and Raji (Fig. 1d), but no significant difference was observed. Significance was evaluated as described in “Methods” section.

### Discussion

#### Antibiotic cytotoxicity assays

Human cell lines HEK293, OVCAR8, and CA46 were evaluated for cytotoxicity in response to increasing concentrations of the antibiotics chloramphenicol, tetracycline, fusidic acid, kanamycin, linezolid, and streptomycin. Cell lines Ramos and Raji were evaluated only against chloramphenicol. Representative results are illustrated in Additional files 1, 2, 3, 4. The cell lines demonstrated varying sensitivities to the different antibiotics. All cell lines treated with fusidic acid and chloramphenicol were sensitive, but CA46 exhibited the lowest sensitivity to chloramphenicol. Only fusidic acid was able to demonstrate complete cell killing. The remaining antibiotics showed either no decrease in viability, or a partial decrease at high concentrations.

The EC_50_ values for the six antibiotics are summarized in Additional file 5. Values for tetracycline, chloramphenicol, and linezolid were estimated because complete cell killing could not be achieved. The EC_50_ values for most antibiotics were well above the therapeutic blood plasma levels in patients (summarized in Table 2) [24]. Only fusidic acid treatment showed EC_50_ values within the therapeutic window of the drug. This suggests that further study of fusidic acid as a cancer therapeutic may be warranted.

**Table 2:** Therapeutic blood plasma levels of antibiotics (calculated from [24])

Antibiotic	Concentration (µM)
Chloramphenicol	15–46
Tetracycline	2–23
Fusidic acid	58–387
Kanamycin	2–52
Linezolid	1–12
Streptomycin	2–69

The ethanol and DMSO solvents used to dissolve chloramphenicol, fusidic acid, and linezolid can be toxic to cells [25, 26]. While ethanol and DMSO may affect cell proliferation, we do not expect that the concentrations of solvent in our experiments influenced our observations. Both solvents were added to cultured cells at concentrations of 1% or less in the antibiotic cytotoxicity assays, and used at 0.003% or less in the combination assays. These concentration are unlikely to have a significant impact on our results [25, 26].

#### Combination cytotoxicity assays

Antibiotics did not significantly alter the sensitivity of HEK293 (Fig. 1a) or OVCAR8 (Fig. 1b) cells to HB21-LR when compared to a control treated only with HB21-LR. CA46 cells were unaffected by five of the antibiotics, but did show significantly enhanced sensitivity to HB21-LR in the presence of chloramphenicol (Fig. 1c). Subsequently, Burkitt’s lymphoma cell lines Raji and Ramos were evaluated for toxicity in combination with chloramphenicol, but no significant effect was observed (Fig. 1d). Representative cytotoxicity assays are shown in Additional files 7, 8, 9, 10.

Antibiotic concentrations used in the combination cytotoxicity assays were identified from antibiotic-alone cytotoxicity assays by selecting high but nontoxic concentrations of each antibiotic (see the vertical dotted line in Additional files 1, 2, 3, 4). Most concentrations tested were above the therapeutic blood plasma level (Table 2) of the six antibiotics, and therefore unlikely to be useful in a clinical setting.

It is noteworthy that CA46 cells were especially insensitive to chloramphenicol. The only significant effect we observed in the combination treatments was CA46 treated with chloramphenicol. For this combination, we employed a concentration (500 µM) that was more than ten times greater than the therapeutic blood plasma concentration of the antibiotic (15–46 µM). The 500 µM concentration of chloramphenicol was also well above the toxic blood plasma concentration of the drug, 77 µM [24]. Raji and Ramos cells were both more sensitive to chloramphenicol than CA46 (Additional files 3, 4, 5), and showed no enhanced cytotoxicity in the combination treatment (Fig. 1).

Fusidic acid was the only antibiotic consistently evaluated at concentrations below the therapeutic blood plasma range of 58–387 µM. Although fusidic acid did not enhance the cytotoxicity of HB21-LR at the concentrations we tested, the low concentration required to diminish cell viability suggests that higher doses might be worth evaluating. Recent studies have suggested that fusidic acid, and derivatives thereof, may have therapeutic utility for the treatment of cancers [27, 28]. We conclude that further investigation into synergy between the six antibiotics evaluated and RITs is not warranted, but it may be useful to explore fusidic acid alone as a treatment for cancer.

## Limitations

- Only the anti-transferrin receptor/PE24 RIT HB21-LR was evaluated. It is not known if RITs with other targets, other forms of PE, or other toxins may behave differently.
- Only six antibiotics were evaluated. Other antibiotics may display different effects.
- Only a single dose of antibiotic was evaluated in combination with HB21-LR. A more extensive evaluation of different combinations could reveal additional effects.
- Only the human cell lines HEK293, OVCAR8, CA46, Raji, and Ramos were evaluated. Other human cell lines may exhibit different responses to treatment.

## Additional files

**Additional file 1.** HEK293 survival in response to antibiotic treatment. Survival of HEK293 cells in response to six antibiotics was evaluated. Each antibiotic was evaluated at least twice. Representative graphs are shown here. Error bars indicate standard error of six replicates. Data were fit to a straight line (streptomycin, kanamycin) or a four-parameter sigmoid function (tetracycline, chloramphenicol, linezolid, fusidic acid). The vertical dotted line indicates the concentration of antibiotic selected for evaluation in combination with HB21-LR (see Table 1).

**Additional file 2.** OVCAR8 survival in response to antibiotic treatment. Survival of OVCAR8 cells in response to six antibiotics was evaluated. Each antibiotic was evaluated at least twice. Representative graphs are shown here. Error bars indicate the standard error of six replicates. Data were fit to a straight line (streptomycin, linezolid, kanamycin) or a four-parameter sigmoid function (tetracycline, chloramphenicol, fusidic acid). The vertical dotted line indicates the concentration of antibiotic selected for evaluation in combination with HB21-LR (see Table 1).

**Additional file 3.** CA46 survival in response to antibiotic treatment. Survival of CA46 cells in response to six antibiotics was evaluated. Each antibiotic was evaluated at least twice. Representative graphs are shown here. Error bars indicate the standard error of six replicates. Data were fit to a straight line (tetracycline, streptomycin, linezolid, kanamycin) or a four-parameter sigmoid function (chloramphenicol, fusidic acid). The vertical dotted line indicates the concentration of antibiotic selected for evaluation in combination with HB21-LR (see Table 1).

**Additional file 4.** Raji and Ramos survival in response to chloramphenicol treatment. Survival of Raji and Ramos cells in response to chloramphenicol was evaluated. Each cell line was evaluated three times. Representative graphs are shown here. Error bars indicate the standard error of six replicates. Data were fit to a four-parameter sigmoid function. The vertical dotted line indicates the concentration of chloramphenicol selected for evaluation in combination with HB21-LR (see Table 1).

**Additional file 5.** Antibiotic cytotoxicity EC_50_ values (mM). The survival of HEK293, OVCAR8, and CA46 cells in response to six antibiotics was evaluated. The survival of Raji and Ramos cells were evaluated in response to chloramphenicol. Each antibiotic tested was evaluated on each cell line at least twice. Where cytotoxicity was observed, data were fit to a four-parameter sigmoid function. The EC_50_ was extracted from the curve fit and is presented in tabular format here. Estimates were taken for those antibiotics where complete cell killing was not achieved. If no toxicity was observed, that is indicated.

**Additional file 6.** Maximum antibiotic concentrations evaluated. The survival of cells was evaluated in response to the antibiotics chloramphenicol, tetracycline, fusidic acid, kanamycin, linezolid, and streptomycin. The maximum concentration of antibiotic tested on cells is shown here.

**Additional file 7.** HEK293 survival in response to antibiotic/RIT combination treatment. Survival of HEK293 cells was evaluated in response to combination treatment with antibiotic and HB21-LR. Antibiotic concentrations are shown in Table 1. Each combination was evaluated at least three times. Representative graphs comparing HB21-LR alone to HB21-LR with antibiotic are shown here. Error bars indicate standard error of six replicates. Data were fit to a four-parameter sigmoid function.

**Additional file 8.** OVCAR8 survival in response to antibiotic/RIT combination treatment. Survival of OVCAR8 cells was evaluated in response to combination treatment with antibiotic and HB21-LR. Antibiotic concentrations are shown in Table 1. Each combination was evaluated at least three times. Representative graphs comparing HB21-LR alone to HB21-LR with antibiotic are shown here. Error bars indicate standard error of six replicates. Data were fit to a four-parameter sigmoid function.

**Additional file 9.** CA46 survival in response to antibiotic/RIT combination treatment. Survival of CA46 cells was evaluated in response to combination treatment with antibiotic and HB21-LR. Antibiotic concentrations are shown in Table 1. Each combination was evaluated at least three times. Representative graphs comparing HB21-LR alone to HB21-LR with antibiotic are shown here. Error bars indicate standard error of six replicates. Data were fit to a four-parameter sigmoid function.

**Additional file 10.** Raji and Ramos survival in response to chloramphenicol/RIT combination treatment. Survival of cells Raji and Ramos cells was evaluated in response to combination treatment with chloramphenicol and HB21-LR. Antibiotic concentrations are shown in Table 1. Each combination was evaluated at least three times. Representative graphs comparing HB21-LR alone to HB21-LR with chloramphenicol are shown here. Error bars indicate standard error of six replicates. Data were fit to a four-parameter sigmoid function.

## Data Availability

Materials that are not commercially available are available from the corresponding author upon request.

## Abbreviations

DMEM: Dulbecco’s Modified Eagle’s Medium
DMSO: dimethyl sulfoxide
PE: *Pseudomonas* exotoxin A
RIT: recombinant immunotoxin
