# Supplementary material for: Evaluating the influence of common antibiotics on the efficacy of a recombinant immunotoxin in tissue culture
Source: BMC Res Notes. 2019 May 27;12:293. doi: 10.1186/s13104-019-4337-6 (PMC6537151; doi:10.1186/s13104-019-4337-6)
Supplement: Supplementary file 1 — Additional file 1. HEK293 survival in response to antibiotic treatment. Survival of HEK293 cells in response to six antibiotics was evaluated. Each antibiotic was evaluated at least twice. Representative graphs are shown here. Error bars indicate standard error of six replicates. Data were fit to a straight line (streptomycin, kanamycin) or a four-parameter sigmoid function (tetracycline, chloramphenicol, linezolid, fusidic acid). The vertical dotted line indicates the concentration of antibiotic selected for evaluation in combination with HB21-LR (see Table 1). [file 13104_2019_4337_MOESM1_ESM.pdf]

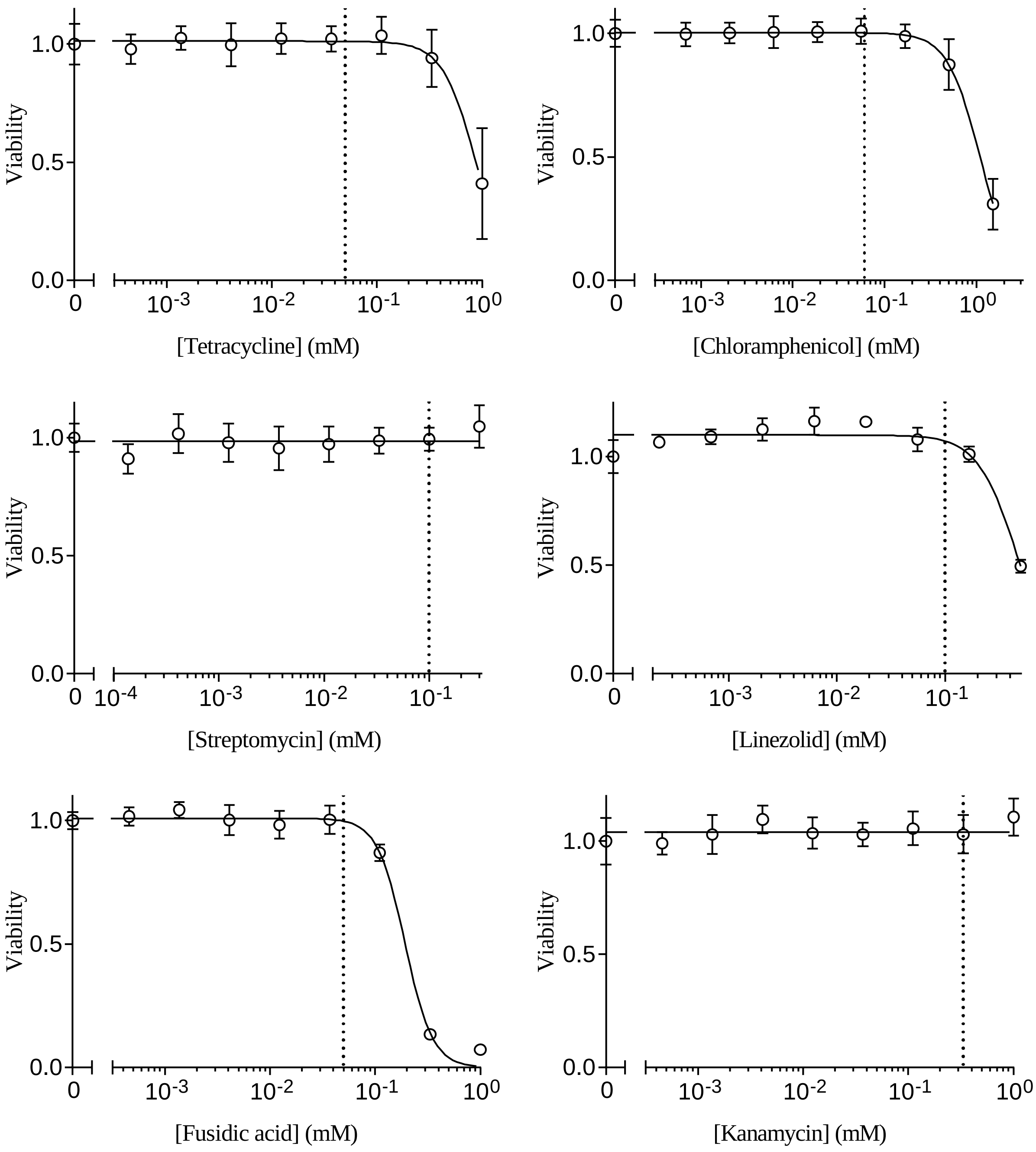

Additional file 1. HEK293 survival in response to antibiotic treatment. Survival of HEK293 cells in response to six antibiotics was evaluated. Each antibiotic was evaluated at least twice. Representative graphs are shown here. Error bars indicate standard error of six replicates. Data were fit to a straight line (streptomycin, kanamycin) or a four-parameter sigmoid function (tetracycline, chloramphenicol, linezolid, fusidic acid). The vertical dotted line indicates the concentration of antibiotic selected for evaluation in combination with HB21-LR (see Table 1).
