# Supplementary material for: Evaluating the influence of common antibiotics on the efficacy of a recombinant immunotoxin in tissue culture
Source: BMC Res Notes. 2019 May 27;12:293. doi: 10.1186/s13104-019-4337-6 (PMC6537151; doi:10.1186/s13104-019-4337-6)
Supplement: Supplementary file 4 — Additional file 4. Raji and Ramos survival in response to chloramphenicol treatment. Survival of Raji and Ramos cells in response to chloramphenicol was evaluated. Each cell line was evaluated three times. Representative graphs are shown here. Error bars indicate the standard error of six replicates. Data were fit to a four-parameter sigmoid function. The vertical dotted line indicates the concentration of chloramphenicol selected for evaluation in combination with HB21-LR (see Table 1). [file 13104_2019_4337_MOESM4_ESM.pdf]

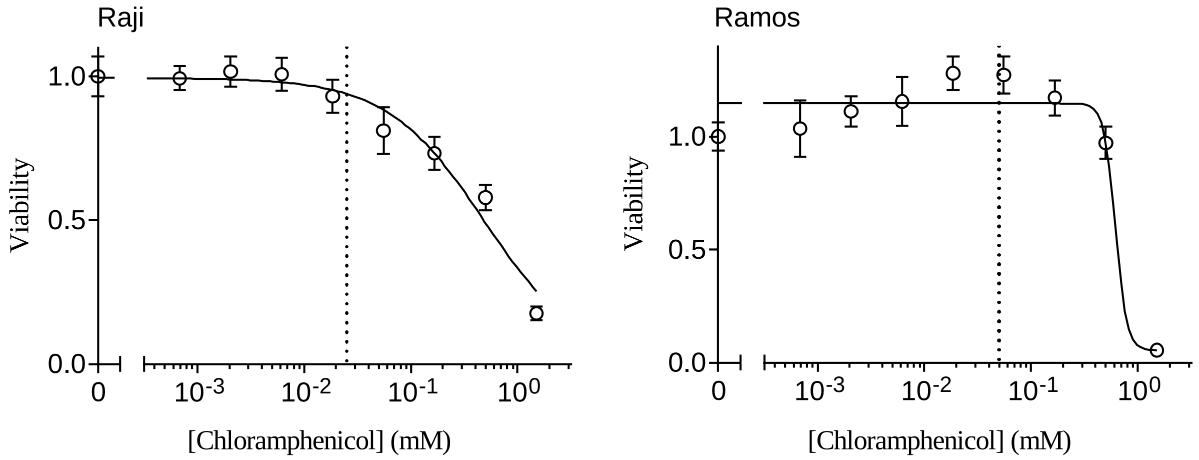

Additional file 4. Raji and Ramos survival in response to chloramphenicol treatment. Survival of Raji and Ramos cells in response to chloramphenicol was evaluated. Each cell line was evaluated three times. Representative graphs are shown here. Error bars indicate the standard error of six replicates. Data were fit to a four-parameter sigmoid function. The vertical dotted line indicates the concentration of chloramphenicol selected for evaluation in combination with HB21-LR (see Table 1).
