# Supplementary material for: Evaluating the influence of common antibiotics on the efficacy of a recombinant immunotoxin in tissue culture
Source: BMC Res Notes. 2019 May 27;12:293. doi: 10.1186/s13104-019-4337-6 (PMC6537151; doi:10.1186/s13104-019-4337-6)
Supplement: Supplementary file 5 — Additional file 5. Antibiotic cytotoxicity EC50 values (mM). The survival of HEK293, OVCAR8, and CA46 cells in response to six antibiotics was evaluated. The survival of Raji and Ramos cells were evaluated in response to chloramphenicol. Each antibiotic tested was evaluated on each cell line at least twice. Where cytotoxicity was observed, data were fit to a four-parameter sigmoid function. The EC50 was extracted from the curve fit and is presented in tabular format here. Estimates were taken for those antibiotics where complete cell killing was not achieved. If no toxicity was observed, that is indicated. [file 13104_2019_4337_MOESM5_ESM.pdf]

Additional file 5. Antibiotic cytotoxicity EC_{50} values (mM).

Antibiotic	HEK293	OVCAR8	CA46	Ramos	Raji
Chloramphenicol	~0.8	~1.0	~1.4	~0.6	~0.7
Tetracycline	~0.6	>1.0	No toxicity observed	-	-
Fusidic Acid	0.171	0.181	0.145	-	-
Kanamycin	No toxicity observed	No toxicity observed	No toxicity observed	-	-
Linezolid	~0.5	No toxicity observed	No toxicity observed	-	-
Streptomycin	No toxicity observed	No toxicity observed	No toxicity observed	-	-
