# Supplementary material for: Evaluating the influence of common antibiotics on the efficacy of a recombinant immunotoxin in tissue culture
Source: BMC Res Notes. 2019 May 27;12:293. doi: 10.1186/s13104-019-4337-6 (PMC6537151; doi:10.1186/s13104-019-4337-6)
Supplement: Supplementary file 6 — Additional file 6. Maximum antibiotic concentrations evaluated. The survival of cells was evaluated in response to the antibiotics chloramphenicol, tetracycline, fusidic acid, kanamycin, linezolid, and streptomycin. The maximum concentration of antibiotic tested on cells is shown here. [file 13104_2019_4337_MOESM6_ESM.pdf]

Additional file 6. Maximum antibiotic concentrations evaluated.

Antibiotic	[Maximum] (mM)
Chloramphenicol	1.5
Tetracycline	1.0
Fusidic Acid	1.0
Kanamycin	1.0
Linezolid	0.5
Streptomycin	0.3
