# Supplementary material for: Evaluating the influence of common antibiotics on the efficacy of a recombinant immunotoxin in tissue culture
Source: BMC Res Notes. 2019 May 27;12:293. doi: 10.1186/s13104-019-4337-6 (PMC6537151; doi:10.1186/s13104-019-4337-6)
Supplement: Supplementary file 10 — Additional file 10. Raji and Ramos survival in response to chloramphenicol/RIT combination treatment. Survival of cells Raji and Ramos cells was evaluated in response to combination treatment with chloramphenicol and HB21-LR. Antibiotic concentrations are shown in Table 1. Each combination was evaluated at least three times. Representative graphs comparing HB21-LR alone to HB21-LR with chloramphenicol are shown here. Error bars indicate standard error of six replicates. Data were fit to a four-parameter sigmoid function. [file 13104_2019_4337_MOESM10_ESM.pdf]

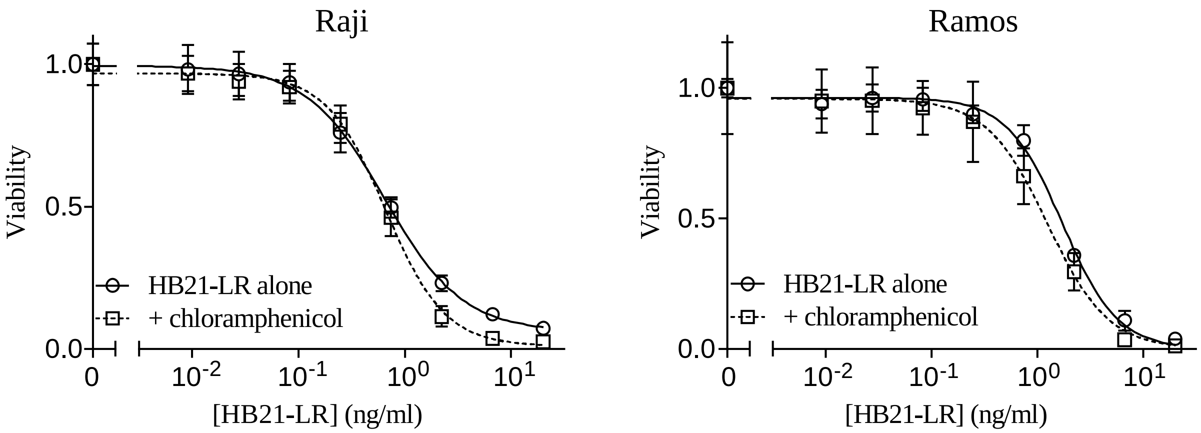

Additional file 10. Raji and Ramos survival in response to chloramphenicol/RIT combination treatment. Survival of cells Raji and Ramos cells was evaluated in response to combination treatment with chloramphenicol and HB21-LR. Antibiotic concentrations are shown in Table 1. Each combination was evaluated at least three times. Representative graphs comparing HB21-LR alone to HB21-LR with chloramphenicol are shown here. Error bars indicate standard error of six replicates. Data were fit to a four-parameter sigmoid function.
